# Livestock trade network: potential for disease transmission and implications for risk-based surveillance on the island of Mayotte

**DOI:** 10.1038/s41598-018-29999-y

**Published:** 2018-08-01

**Authors:** Younjung Kim, Laure Dommergues, Ali Ben M’sa, Philippe Mérot, Eric Cardinale, John Edmunds, Dirk Pfeiffer, Guillaume Fournié, Raphaëlle Métras

**Affiliations:** 1 Veterinary Epidemiology, Economics and Public Health group, Department of Pathobiology and Population Sciences, The Royal Veterinary College, Hatfield, UK; 2 Centre for Applied One Health Research and Policy Advice, College of Veterinary Medicine and Life Sciences, City University of Hong Kong, Kowloon, Hong Kong SAR, China; 3 GDS Mayotte-Coopérative Agricole des Eleveurs Mahorais, Coconi, Mayotte France; 4 Direction de l’Alimentation, de l’Agriculture et de la Forêt de Mayotte, Mamoudzou, France; 5 Centre de Coopération Internationale en Recherche agronomique pour le Développement (CIRAD), UMR ASTRE, Cyroi platform, Sainte Clotilde, La Réunion, France; 6 Centre de Coopération Internationale en Recherche Agronomique pour le Développement (CIRAD), UMR ASTRE “Animals, Health, Territories, Risks, and Ecosystems”, Montpellier, France; 7 Centre for the Mathematical Modelling of Infectious Diseases, Department of Infectious Disease Epidemiology, London School of Hygiene & Tropical Medicine, London, UK; 8 ASTRE, Université de Montpellier (I-MUSE), CIRAD, Institut National de la Recherche Agronomique, Montpellier, France

## Abstract

The island of Mayotte is a department of France, an outermost region of the European Union located in the Indian Ocean between Madagascar and the coast of Eastern Africa. Due to its close connection to the African mainland and neighbouring islands, the island is under constant threat of introduction of infectious diseases of both human and animal origin. Here, using social network analysis and mathematical modelling, we assessed potential implications of livestock movements between communes in Mayotte for risk-based surveillance. Our analyses showed that communes in the central region of Mayotte acted as a hub in the livestock movement network. The majority of livestock movements occurred between communes in the central region and from communes in the central region to those in the outer region. Also, communes in the central region were more likely to be infected earlier than those in the outer region when the spread of an exotic infectious disease was simulated on the livestock movement network. The findings of this study, therefore, suggest that communes in the central region would play a major role in the spread of infectious diseases via livestock movements, which needs to be considered in the design of risk-based surveillance systems in Mayotte.

## Introduction

The South Western Indian Ocean islands, including the Comoros, La Reunion, Madagascar, Mayotte and Seychelles, are connected to each other and to the African mainland by the movement of people and animals^1^. Among these islands, Mayotte, located in the Northern Mozambique Channel, is highly connected to its neighbouring territories. In particular, illegal import of livestock from the Comoros is not uncommon^2^, making the island vulnerable to the introduction of infectious diseases affecting animals and/or humans. Mayotte has recently suffered from the introductions of Dengue, Chikungunya, and Rift Valley fever (RVF) viruses, the latter probably resulting from the introduction of a lineage from the African continent via illegally imported animals from the Comoros^1–3^. Moreover, its livestock population is under constant threat from recent outbreaks of Peste des Petits Ruminants (PPR) in the Comoros and high endemicity of Foot-and-Mouth disease (FMD) and lumpy skin disease in Eastern Africa^4–6^, increasing the potential for Mayotte to serve as an entry point of these diseases into Europe, as an outermost region of the European Union.

The livestock population in Mayotte was estimated to be approximately 20,000 for cattle and 13,000 for small ruminants^7^. In addition to the national surveillance programmes for brucellosis and bovine tuberculosis, an animal disease surveillance system (SESAM, Système d’Epidémiosurveillance Animale à Mayotte) has been in operation in Mayotte since 2009. Although it depends on both active (e.g. RVF serosurveys) and passive (e.g. abortion and mortality reports by farmers) surveillance components, the detection of exotic livestock disease heavily relies on active surveillance since reporting livestock disease by Mayotte farmers may not always be systematic (Dommergues, personal communication). Given this situation, the effective and efficient allocation of limited resources has particular importance since active surveillance requires considerable resources. Risk-based surveillance could achieve this by incorporating prior information about its target population, derived from epidemiological studies, into the design of traditional surveillance^8^.

The contact network can be used to infer the underlying transmission network since it influences how disease transmits among the population combined with host infection status^9^. Among different types of contacts, livestock movements constitute major routes for the spread of infectious livestock disease^10^. For example, the long-range movement of sheep in combination with local transmission resulted in the FMD epidemic in the UK in 2001^10,11^. Livestock movements could, therefore, provide insight into the structure of the underlying transmission network and thus allow early detection and more effective management of infectious disease^12^. To do this, social network analysis and mathematical modelling have been employed widely to describe contact patterns of livestock movements and analyse their potential use for designing disease control strategies^12^.

This study aimed at identifying the network characteristics of livestock movements in Mayotte and assessing their potential implications for risk-based surveillance. First, these data were presented as a network of administrative communes connected by livestock movements and characterised by social network analysis at both the global and commune levels. Second, we assessed the time from the introduction of an exotic livestock disease into the island to the infection of individual communes by stochastic simulations. Finally, to assess the validity of the inferences from our analyses, we compared RVF IgG seroprevalence between clusters of communes that were classified based on their similarity in the network position (i.e. structural equivalence) over the study years, using longitudinal RVF IgG seroprevalence data^2^.

## Methods

### Data

Data on livestock movements that occurred from 2007–2014 were collected from two sources: the official farm registry of the Chambre de l’Agriculture, de la Pêche et de l’Aquaculture de Mayotte (CAPAM), and the records of livestock moved by truck by the Coopérative des Eleveurs de Mayotte. While the official farm registry included the date and the farms of origin and destination for each movement of each animal, the truck dataset only contained information about the number of animals moved between communes on a given date. We merged the two datasets into one, which resulted in 3505 livestock movement records between communes (detailed in Supplementary Material A).

### Network Characteristics

All livestock movements that occurred in a study year, that is from July to June of the following year, were aggregated to draw seven yearly static networks. Study years were used to capture the rainy season as a whole (November-March) since it was considered to influence livestock movements. The networks were directed and weighted, with the 17 communes as nodes and livestock movements as edges. The number of livestock moved from commune *i* to commune *j* was the weight of a directed edge from *i* to *j*.

First, the large-scale structure of each yearly network was assessed by the density (i.e. the proportion of potential edges present in a network), giant strongly and weakly connected components (GSCC, GWCC), average path length and clustering coefficient^13,14^. The GSCC and GWCC were to assess the lower and upper limit of the maximum epidemic size, respectively^14^. The average path length and clustering coefficient of the observed networks were compared to the metrics of random networks to assess whether yearly networks exhibited small-world properties^15^ (detailed in Supplementary Material B). Between all pairs of yearly networks, the correlation in the distributions of edges was assessed through a quadratic assignment procedure (QAP)^16^.

Second, in each yearly network, the similarity in the position of nodes was assessed by exploring their structural equivalence. Euclidean distance was used as a measure of structural equivalence to account for not only the presence of livestock movements but also the number of animals moved between communes^17^. That is, two communes were considered structurally equivalent if they received and sent the same number of animals from, and to, the same communes, excluding the two communes being compared^17^. As in other network characteristics, livestock movements within communes were not considered when structural equivalence was assessed. To identify clusters of communes with similar livestock movement patterns, communes were then classified into two clusters based on Euclidean distances computed for all pairs of communes. These structurally equivalent clusters were defined using Ward’s criterion^18,19^ at the point where two clusters resulted in the total minimum within-cluster variance. The statistical significance of commune partitions into structurally equivalent clusters was explored by comparing the edge density and the number of livestock moved within and between clusters between observed and permuted networks.

Third, communes’ importance was assessed with the following centrality measures: in- and out-strength, betweenness and closeness (detailed in Supplementary Material B). The correlation between those centrality measures was examined by the Kendall rank correlation test.

Finally, the associations between in- and out-strength of a commune and its human and livestock population sizes were assessed by linear regression, accounting for the non-independence of network data by using a permutation test^20^. The association between the number of livestock moved between communes and the shortest road distance was explored using the QAP regression tests^21,22^, based on a semi-partialing permutation method^16,23^. Road distances between communes were calculated using the x-y coordinates of the centre of the largest town in each commune as a geographical reference point^24^.

### Dynamic Epidemic Simulation

#### Baseline model

Epidemic spread through livestock movements was simulated. The spread of a hypothetical exotic infectious disease between the communes of Mayotte through the movements of livestock was assessed by stochastic simulations. At the start of a simulation, all communes were susceptible. The infection was seeded in a randomly selected commune and on a random day of the study period (2007–2014). Daily livestock movements between communes were then replayed as recorded in the dataset until the end of the study period. The infection status of susceptible commune *j* upon receiving ${W}_{i,j,d}$ animals from commune *i* on day *d* was simulated by a Bernoulli trial with ${P}_{i,j,d}$, the probability that commune *j* became infected, as the probability of success. ${P}_{i,j,d}$ was defined as:

$${P}_{i,j,d}=\{\begin{array}{c}1-{(1-{p}_{\inf })}^{{W}_{i,j,d}},{\rm{if}}\,{\rm{commune}}\,{\rm{i}}\,{\rm{was}}\,{\rm{infected}}\\ \,\,\,\,\,\,\,\,\,\,\,\,\,\,\,\,0\,\,\,\,\,\,\,\,\,\,\,\,,{\rm{if}}\,{\rm{commune}}\,{\rm{i}}\,{\rm{was}}\,{\rm{not}}\,{\rm{infected}}\end{array}$$

where ${p}_{\inf }$ was the probability that an animal from an infected commune was infectious and transmitted the disease to other animals in the susceptible commune where it was introduced. Infected communes remained infected until the end of a simulation. We generated 20,000 epidemic simulations for each value of ${p}_{\inf }$: 0.1, 0.5 and 0.9, corresponding to low, medium and high disease transmission scenarios, respectively.

The results of the epidemic simulation were analysed in the following ways. First, we discarded simulations in which less than half of the communes were infected as we only wanted to consider simulations that resulted in large epidemics. Second, to assess the potential of communes to act as sentinels during large epidemics, we computed for each simulation and each commune the time from the day when the infection was seeded to the day when the commune was infected (‘time-to-infection’), except for the commune in which the infection was seeded. For each commune, we then assessed the distribution of time-to-infection values using the median as the measure of central tendency.

#### Model accounting for potential bias

We assessed the potential bias in the model outcome that could arise from the data. First, recall bias in the reported movement dates was possible. It could change the temporal sequence of animal movements and, therefore, epidemic patterns. We, therefore, assessed the impact of modifying those reporting dates on the simulated epidemics. We assumed that 10 to 30% of the livestock movements occurred +/− one month around their reported dates. The following steps were repeated for each simulation and reported livestock movement of each dataset. We first simulated whether a livestock movement actually occurred on its reported date through a Bernoulli trial with the parameter being drawn from a Uniform distribution U[0.7, 0.9]. If the trial was successful, the livestock movement occurred on its reported date. If not, the date was simulated by adding to the reported date a rounded random number drawn from a Normal distribution (μ = 0, σ^2^ = 100) truncated between −30 and 30. We then merged the two datasets, accounting for possible overlap between them. When there were livestock movements that occurred on the same date between the same communes in both datasets, we randomly selected the number of livestock movements among its possible values. For example, if there were 5 and 3 movements in the official and truck datasets, respectively, one value was randomly selected from integer values between 5 and 8.

Finally, some of the simulations finished before infecting all communes since every simulation ended on the last day of the study period regardless of when the infection was seeded. To assess the extent of bias from this, we considered that the time-to-infection for communes which did not become infected during the course of a given simulation was equal to (1) the [length of the simulation +1 day] or (2) [infinity]. The actual time-to-infection for these communes, therefore, lay between these two bounds.

### RVF Seroprevalence and network structure

To explore the validity of our analyses, we used longitudinal RVF IgG seroprevalence data from Mayotte^2^. We assessed whether the distribution of RVF IgG seroprevalence across the island could be associated with the movements of animals by comparing annual RVF IgG seroprevalence patterns between structurally equivalent clusters, with a particular focus on an RVF epidemic between 2008–2010^3^. More specifically, annual RVF IgG seroprevalence in clusters of communes that were classified based on communes’ structural equivalence was estimated. The seroprevalence ${p}_{c,y}$ in a commune cluster *c* (central or outer) on year *y* was:

$${p}_{c,y}=\frac{\sum _{i,i\in c}{P}_{i,y}}{\sum _{i,i\in c}{N}_{i,y}}$$

with ${N}_{i,y}$ and ${P}_{i,y}$ being the total number of animals and the number of positive animals sampled on commune *i* in year *y*, respectively. The 95% confidence intervals were obtained by accounting for clustering at herd level, using varbin function of the aod package^25^ in R^26^. We further explored whether the between-cluster difference observed in RVF IgG seroprevalence could be explained by vector-related transmission. For this, we used Normalised Difference Vegetation Index (NDVI) as a proxy of mosquito abundance since it reflects the level of vegetation and water presence, i.e. conditions promoting mosquito proliferation^27^.

### Data availability

The official and truck datasets are available from Supplementary Information S1 and S2, respectively. Commune IDs and their geographic coordinates are available from Supplementary Information S3. Model codes for epidemic simulation are available from Supplementary Information S4 (original model) and S5 (model accounting for potential recall bias) online as R scripts.

## Results

### Network Characteristics

**Figure 1:**
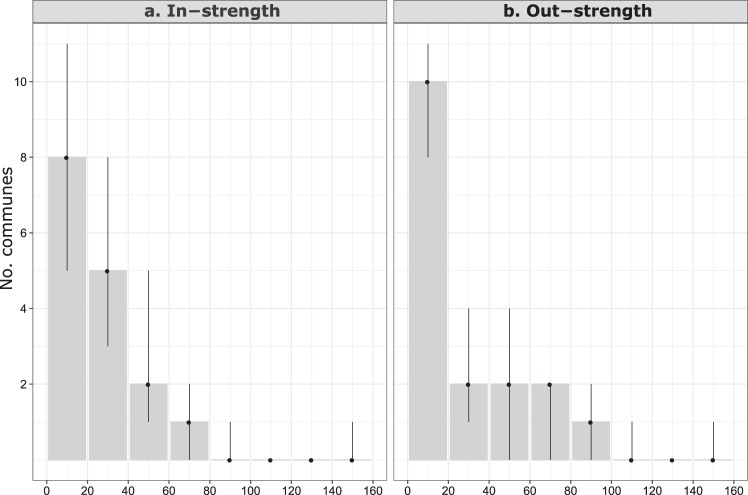
In- and out-strength distributions of the livestock movement network. The in- and out-strength were aggregated over the study period, and their median values were presented as frequency distributions. The low and high ends of the vertical bars corresponded to the minimum and maximum value, respectively.

During the study period, the number of between-commune livestock movements peaked in 2009 (n = 712, 22.3%), whereas it was the lowest in 2014 (n = 350, 11.0%). The density of yearly networks ranged between 0.40 and 0.52, and the total number of livestock movements per year between 350 and 712. The number of livestock moved in (in-strength) and out (out-strength) of a commune ranged from 2 to 142, and from 0 to 155, respectively, and their distributions were right-skewed (Fig. 1). All yearly networks were connected via livestock movements, with the GWCC always including the 17 communes, and the GSCC either 16 or 17 communes. The networks were strongly correlated between all pairs of yearly networks, suggesting that the overall structure of the livestock movement network in Mayotte was stable over the study years; the correlation statistic between any two networks was significantly higher than those between the permuted networks (all *p*-values < 0.001).

All yearly networks showed a high level of clustering, which was significantly higher than in random networks (Supplementary Fig. S1). However, they did not seem to exhibit small-world properties as the average path length was longer than for random networks, in all but one study year (Supplementary Fig. S2).

**Figure 2:**
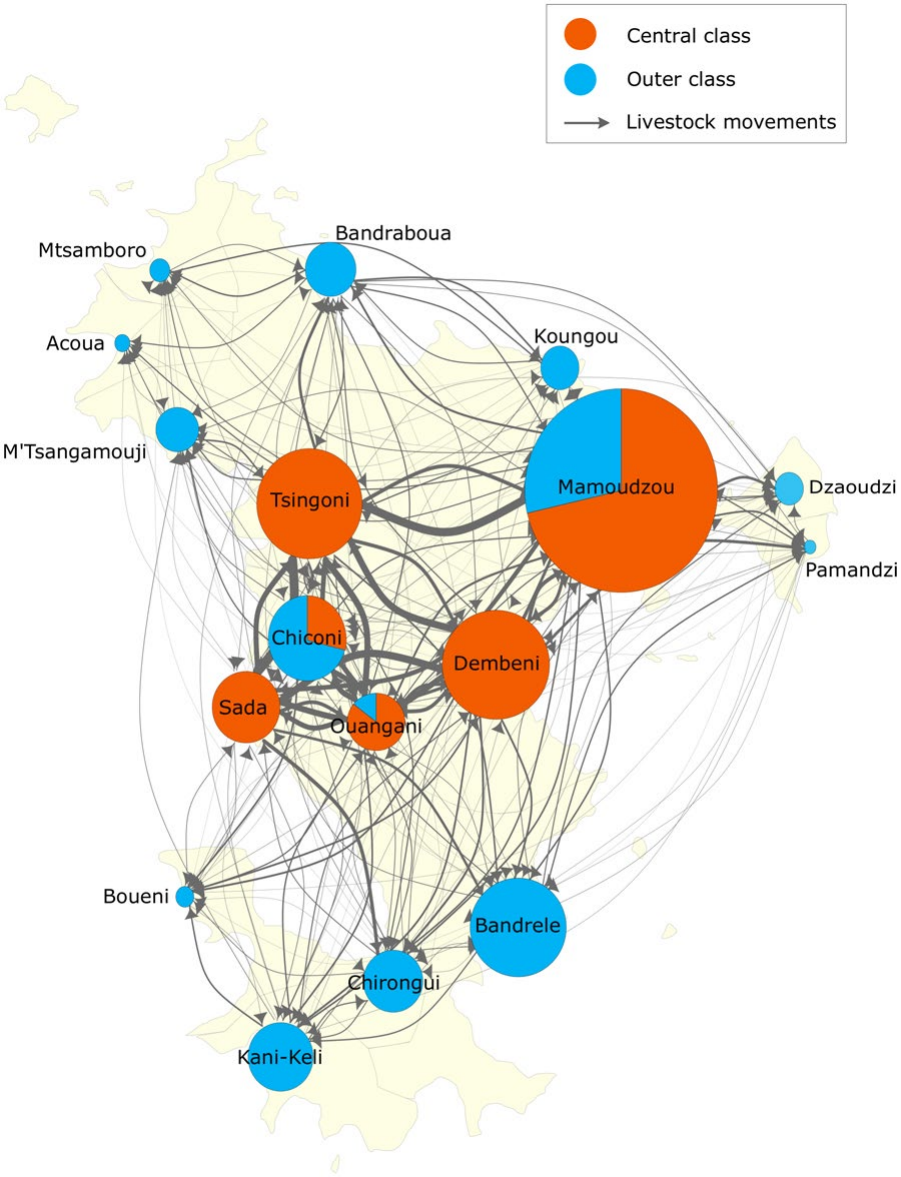
The livestock movement network in Mayotte from 2007 to 2014. Nodes and edges corresponded to communes and livestock movements between communes, respectively. The size of the nodes represented the number of livestock in the communes in 2010. The arrow and width of the edges represented the direction and the total number of livestock movements between communes over the study years, respectively. The nodes were expressed as pie charts, showing the proportion of communes’ structurally equivalent cluster over the study years.

**Figure 3:**
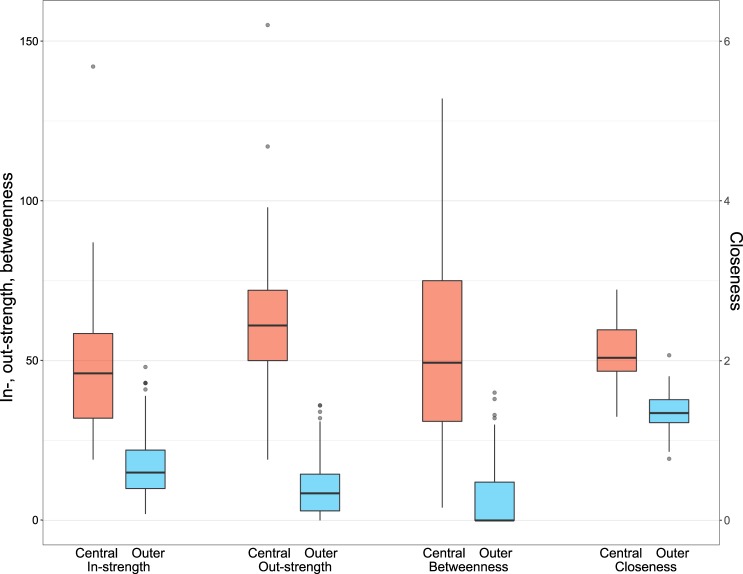
In-strength, out-strength, betweenness (left y-axis) and closeness (right y-axis) by structurally equivalent cluster over the study years.

Each yearly network was partitioned into two structurally equivalent clusters, with this partition being correlated with the geographical location of the communes (Fig. 2). Communes in the central region of the island of Mayotte tended to be classified as one cluster (red in Fig. 2), and communes in the outer region as another (blue in Fig. 2); communes in the former cluster were referred to as central communes and those in the latter cluster as outer communes. Cluster composition was stable over the study period, with the membership of only 3 communes varying over the study period, whereas the other 14 communes were always either central (n = 3) or outer (n = 11) (Fig. 2); when presenting results from the yearly networks in aggregate, communes were re-classified as central communes if they were classified as central communes in more than half the yearly networks, otherwise as outer communes. Node connectedness greatly varied according to their membership. Nodes’ centrality measures tended to be higher in central than outer communes (Fig. 3). More animals were moved between central than between outer communes, and from central to outer communes than from outer to central communes, except for one study year (Supplementary Fig. S3). In every study year, the number of livestock movements between central communes (or between outer communes) was significantly higher (or lower) in the observed network than in the permuted networks. However, there was no significant difference in the number of livestock movements from central to outer communes (or from outer to central communes) between the observed and permuted networks. Furthermore, in every study year, almost all possible edges were realised between central communes (i.e. density = 1), whereas less than a third between outer communes (Supplementary Fig. S4).

Finally, in each of the study years, out-strength was associated with the size of the livestock population (Supplementary Table S1); on average, communes sent out 1–3 more animals as their livestock population increased by 100 animals. In-strength also showed positive correlations, but there was no statistical significance in all but one study year (July 2011–June 2012). The size of the human population had no significant association with in- and out-strength in most study years. Finally, QAP regression tests showed that the number of livestock moved between two communes increased as the distance between them decreased (Supplementary Table S2).

### Dynamic Epidemic Simulations

**Figure 4:**
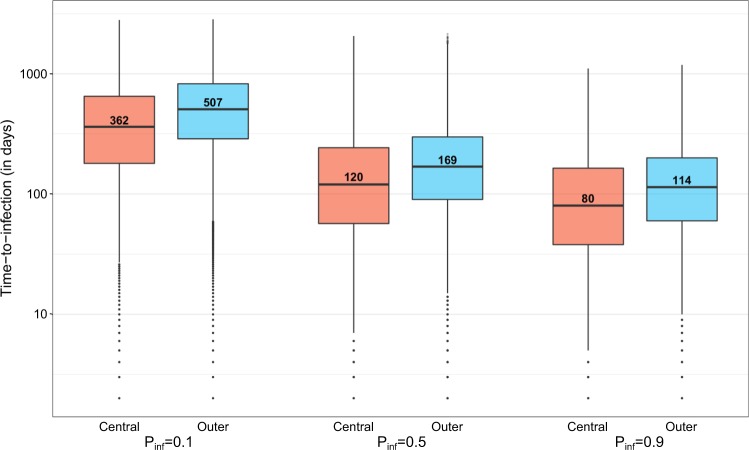
Time from disease incursion to commune infection (in days) aggregated by structurally equivalent cluster (Baseline model). For the three values of ${p}_{\inf }$ tested (0.1, 0.5, 0.9), the number of days from disease incursion to commune infection was aggregated by structurally equivalent cluster (central and outer communes) and plotted in the log scale (y-axis). Numbers in boxplots corresponded to the median values.

**Figure 5:**
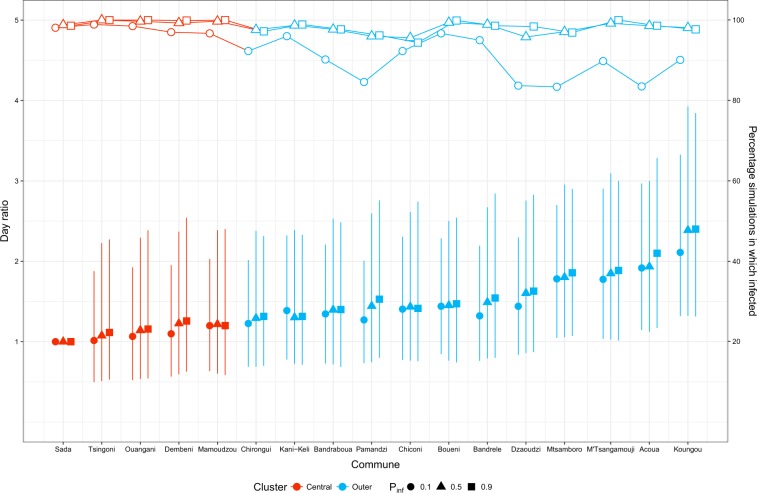
Relative time from disease incursion to infection of individual communes, using the commune of Sada as baseline (left y-axis), and percentage of simulations in which the commune was infected (right y-axis) (Baseline model). The time from disease incursion to commune infection was compared for each commune using the commune of Sada as a baseline. The median, and 1^st^ and 3^rd^ quartiles of individual communes were compared with the median of Sada and presented by filled points and vertical lines. For each commune, the percentage of simulations in which the commune was infected was presented by hollow points and horizontal lines. Different point shapes and colours represented different ${p}_{\inf }$ values and structurally equivalent clusters, respectively.

For the three values of ${p}_{\inf }$ tested (0.1, 0.5, 0.9), the median number of days from disease incursion to commune infection was about 1.4 times lower for central than outer communes (Fig. 4). Less than 3 months were needed for diseases with a high transmission potential to reach central communes (${p}_{\inf }$: 0.9, median: 80 days), whereas it took almost a year for diseases with a low transmission potential (${p}_{\inf }$: 0.1, median: 362 days). On average, the commune of Sada was infected the earliest for all values of ${p}_{\inf }$ and therefore was used as a baseline commune when computing the relative time from disease incursion to commune infection. The relative time did not differ much among central communes but was more variable for outer communes (Fig. 5). In addition, as ${p}_{\inf }$ decreased, the relative time from disease incursion to commune infection (filled points in Fig. 5) and the likelihood of commune infection during simulations (hollow points in Fig. 5) decreased in most communes, especially in outer communes. For the three values of ${p}_{\inf }$ tested, accounting for potential recall bias in the reported dates and possible overlap between the two datasets resulted in almost the same time-to-infection (Supplementary Figs S5 and S6). Finally, after accounting for communes which were not infected during simulations, the outer communes’ time-to-infection slightly increased for ${p}_{\inf }$ = 0.1, while it remained almost unchanged for central communes (Supplementary Table S3).

### RVF seroprevalence and network structure

**Figure 6:**
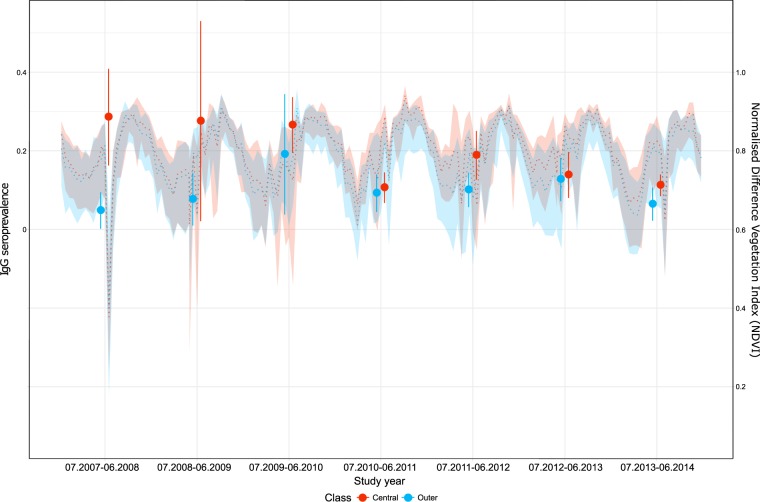
RVF IgG seroprevalence and normalised difference vegetation index (NDVI) by structurally equivalent cluster. Solid vertical lines represented the 95% confidence intervals of RVF IgG seroprevalence of each study year, with points representing the means. Dashed lines represented the medians of NDVI at the 16-day interval, with their background colour representing the range of the 1^st^ and 3^rd^ quantiles. NDVI data were extracted from the Moderate Resolution Imaging Spectroradiometer Terra satellite at 250 m spatial and 16-day temporal resolution^29^.

During the RVF epidemic (2008–2010), RVF IgG seroprevalence was higher in central than outer communes (Fig. 6). The difference between central and outer communes was marked in 2008 when RVF seroprevalence was 5.9 times higher in central than outer communes. However, it became smaller as RVF seroprevalence in outer communes gradually increased between 2009–2010. A similar pattern was also observed after the epidemic period; in central communes, RVF seroprevalence increased in 2012 with decreases in the following study years. In contrast, in outer communes, RVF seroprevalence remained low in 2012 and showed a slight increase in 2013. During the epidemic period, there was little difference in NDVI between central and outer communes.

## Discussion

In the livestock movement network, central communes were closely connected between themselves and to outer communes, whereas outer communes maintained large network distances between themselves or to central communes. These patterns were statistically significantly associated with the number of livestock and the road distance between communes. In the epidemic simulation, central communes tended to be infected earlier than outer communes upon the introduction of exotic livestock disease. Finally, RVF IgG seroprevalence was higher in central than outer communes during the previous epidemic.

Our network analyses showed that central communes acted as a hub in the livestock movement network. Dense connectivity between central communes and from central to outer communes resulted in a higher clustering coefficient than random networks. However, outer communes had relatively sparse connectivity between themselves and to central communes, with the shortest path between outer communes being mostly mediated via central communes. This resulted in a longer average path length than random networks, which contrasted with small-world properties^15^. Under this network structure, an epidemic would have a different pattern in the early stage depending on where a livestock disease is first introduced. If the disease is first introduced into an outer commune, it may be limited within the commune or spread towards central communes rather than the other outer communes. However, once the disease affects central communes, a rapid and widespread epidemic is possible due to dense connectivity between central communes and from central to outer communes. Moreover, the consequence of an epidemic would be more severe when central communes are affected, given a larger livestock population being present in central than outer communes.

Next, the epidemic simulation showed that the difference in the absolute number of days to commune infection decreased sharply as ${p}_{\inf }$ increased. In particular, the difference between central and outer communes also decreased, showing the potential for a rapid and widespread epidemic upon on the introduction of highly transmissible livestock diseases, such as FMD or PPR. Considering that these diseases are endemic in Eastern Africa, surveillance should be reinforced with particular attention to the movement of animals coming from this area.

The epidemic simulation also showed that central communes tended to be infected earlier than outer communes regardless of where the infection was seeded. The difference between central and outer communes could be larger than observed since the epidemic simulation did not account for within-commune factors; a commune was assumed to be infected at the same level as other infected communes as soon as it received an infected animal. However, communes may have different within-commune infection dynamics. In fact, central communes tended to have not only larger livestock and human populations but also more livestock movements within themselves than outer communes. The prevalence of infection may, therefore, be higher in central than outer communes.

We were able to compare the results of our analysis with RVF seroprevalence data as the data available covered most of the study period. In addition, although RVF is a vector-borne disease primarily transmitted to domestic ruminants via mosquito bites, movements of livestock have been suggested as one of the main factors involved in RVF dynamics^2,28^. Last and most importantly, an RVF epidemic between 2008–2010 provided a unique opportunity to assess whether Mayotte communes experienced different epidemic patterns depending on their network properties. The observed trend in RVF seroprevalence may suggest that the actual transmission between communes was shaped rather by livestock movements, assuming that the climate conditions for vectors and animal exposure to the vectors were similar across the whole island. Our study observed that average NDVI was similar in both central and outer communes during the epidemic. Considering also that ruminant livestock in Mayotte are raised outdoors in herds of small sizes^2^, their exposure to mosquito bites might have been similar across the island, and their density might have been too low to have an impact on density-dependent RVF transmission. Under these conditions, the introduction of infected animals might have played a major role in the spread of RVF. The trend of RVF seroprevalence being high in central communes throughout the epidemic period may suggest that these communes acted as a hub for RVF spread via livestock movements. In particular, RVF seroprevalence reached its peak in central communes in the first year of the epidemic, which could be explained by the findings of the network analyses that almost all possible edges were realised between central communes in every study year. That is, RVF might have spread rapidly within central communes at the beginning of the epidemic and then towards outer communes. Data on other diseases, should they become available, could be used to further assess the validity of findings from our analysis.

This study had some limitations. First, our analyses were based on merged data from two sources of livestock movement data collected differently. For the official dataset, livestock movements were recorded when farmers reported to CAPAM in person that they had sent out or received livestock. For this reason, some farmers reported the movement of an animal when they visited CAPAM long after the actual day of the movement. It was, therefore, possible that some of the recorded movement dates were affected by recall bias. However, its impact on the study results should be considered minimal given that our network analyses were performed on yearly static networks. Also, although the epidemic simulation used daily livestock movements, its outcome would have been influenced mainly by the overall flow of livestock movements between communes rather than their actual movement date, as shown in the model which accounted for possible bias. Second, livestock movements might have been underreported in the official dataset. In particular, livestock movements to/from outer communes might have been underreported more than those to/from central communes due to their more difficult access to CAPAM, located in Ouangani, in the centre of the island. In addition, some livestock movements, especially those of small ruminants, might not have been recorded in the truck dataset being moved by other drivers or farmers themselves. To minimise information bias and improve the representativeness of our analyses, we merged livestock movements from both datasets assuming that they complement each other.

Our study has significant implications for the design of risk-based surveillance in Mayotte. The main findings suggest that upon the introduction of exotic livestock disease, central communes are more likely to be infected early than outer communes, and that there is potential for a large epidemic. Considering that the probability of disease occurrence and its consequences are the two main elements in the risk assessment^8^, our study provides valuable information for the design of risk-based surveillance. For example, surveillance could adopt risk-based sampling by stratifying central and outer communes into high- and low-risk groups, respectively. Targeted biosecurity measures could also be applied on livestock movements between and to central communes to minimise the size of an epidemic.

In conclusion, communes in the central region of Mayotte would play a major role in the spread of livestock diseases. A widespread epidemic is likely due to their high connectivity between themselves and to communes in the outer region. Surveillance efforts, therefore, need to be focused on central communes to achieve the timely detection of disease occurrence and minimise its consequences.

## Electronic supplementary material

Supplementary Material

Supplementary Information 1

Supplementary Information 2

Supplementary Information 3

Supplementary Information 4

Supplementary Information 5
