# Supplementary material for: Livestock trade network: potential for disease transmission and implications for risk-based surveillance on the island of Mayotte
Source: Sci Rep. 2018 Aug 1;8:11550. doi: 10.1038/s41598-018-29999-y (PMC6070536; doi:10.1038/s41598-018-29999-y)
Supplement: Supplementary file 1 — Supplementary Material [file 41598_2018_29999_MOESM1_ESM.pdf]

Livestock trade network: potential for disease transmission and implications for risk-based surveillance on the island of Mayotte

Younjung Kim[1,2]*, Laure Dommergues[3], Ali Ben M'sa[3], Philippe Mérot[4], Eric Cardinale[5,6], John Edmunds[7], Dirk Pfeiffer[1,2], Guillaume Fournié[1]$, Raphaëlle Métras[6,7,8]$

* Correspondence to younjung.kim@my.cityu.edu.hk

$ These authors share the last authorship

1 Veterinary Epidemiology, Economics and Public Health group, Department of Pathobiology and Population Sciences, The Royal Veterinary College, Hatfield, UK

2 Centre for Applied One Health Research and Policy Advice, College of Veterinary Medicine and Life Sciences, City University of Hong Kong, Kowloon, Hong Kong SAR

3 GDS Mayotte-Coopérative Agricole des Eleveurs Mahorais, Coconi, Mayotte, France

4 Direction de l'Alimentation, de l'Agriculture et de la Forêt de Mayotte, Mamoudzou, France

5 Centre de Coopération Internationale en Recherche agronomique pour le Développement (CIRAD), UMR ASTRE, Cyroi platform, Sainte Clotilde, La Réunion, France

6 Centre de Coopération Internationale en Recherche Agronomique pour le Développement (CIRAD), UMR ASTRE "Animals, Health, Territories, Risks, and Ecosystems", Montpellier, France

7 Centre for the Mathematical Modelling of Infectious Diseases, Department of Infectious Disease Epidemiology, London School of Hygiene & Tropical Medicine, London, UK

8 ASTRE, Université de Montpellier (I-MUSE), CIRAD, Institut National de la Recherche Agronomique, Montpellier, France

Supplementary Material

Supplementary Method A. Data Cleaning

The official farm registry of the CAPAM (hereafter referred to as the ‘official dataset’) and the records of the CoopADEM (hereafter referred to as the ‘truck dataset’) were in different formats and at different resolutions; recorded at herd level in the official dataset and at commune level in the truck dataset. First, the official dataset consisted of two parts: exit and entry records. The exit records detailed information on animals that left, the herds that animals left, the dates when animals left at a day resolution. The entry records contained the corresponding entry information. After matching the exit and entry records by animal and herd IDs, we rearranged the official dataset in order of movement date to construct a daily sequence of movements for individual animals. We then removed movements from the dataset that might not reflect direct movements between two herds. Reported exit and entry dates could differ from the actual dates since farmers had to visit the CAPAM in person to report movements. Therefore, we defined that a movement was valid if the difference between its exit and entry dates was equal to or less than 180 days, which is the usual length of one season in Mayotte. In contrast, in the truck dataset, exit and entry information were recorded in one dataset. A daily sequence of movements could not be created since animal IDs were not available in the truck dataset. In addition, although movement dates were recorded at a day resolution in the official dataset, origin and destination of movements in the truck dataset were recorded at commune level, not at herd level. We, therefore, decided to perform the analyses at commune level.

In the official dataset, 3,087 movements were identified involving 2,815 different animals. 48.4 percent of the movements (1,493 movements) occurred between communes, and the rest within communes. The exit and entry dates were identical for 2,795 movements (90.5%). The difference between the exit and entry dates was greater than zero and less than 31 days for 189 movements (6.1%). When the exit and entry dates of a given movement were not identical, we assumed that the movement occurred on their median date. One movement was identified for 2,577 animals, whereas two, three and four consecutive movements were identified for 207, 28 and three animals, respectively. On the other hand, the truck dataset comprised a list of 2,207 livestock movements. 91.2 percent of the movements (2,012 movements) occurred between communes. Between 2007

and 2014, the number of recorded movements peaked in 2008 in both datasets (18.7% in the official dataset and 20.2% in the truck dataset). However, while the number of livestock movements in the official dataset showed an increasing trend after a sharp drop in 2009, the number of livestock movements in the truck dataset continued to decrease after 2008 (Supplementary Fig. S7 online).

We assumed that livestock movements of the two datasets represented the same movements if their day of movement, commune origin and destination were the same. Little overlap existed between the two datasets. Indeed, only 0.95 percent of all livestock movements appeared to represent the same movements when matched by calendar day, although this figure increased to 2.07, 5.62 and 17.17 percent when matched by calendar week, month and year, respectively. Therefore, before exploring the livestock movement networks, we merged the official and truck datasets without allowing any overlap between the two datasets.

When livestock movements were aggregated by dry and rainy seasons, the seasonal networks did not show distinct seasonal patterns but rather showed patterns similar to those observed in the yearly static networks. We, therefore, presented results from the yearly static networks.

Supplementary Method B. Network analyses

The network analyses were conducted in the statistical computing environment R[1]. The density, giant strongly connected component and weakly connected components were computed using the *igraph*[2] package. The clustering coefficient and average path length were computed for weighted and binary versions of the networks using the *tnet*[3] package. When accounting for edge weights, Dijkstra[4]'s algorithm was used to compute path lengths. To ensure that yearly networks were comparable, each edge weight was divided by its average over a given network. For the clustering coefficient, we assigned each triad the average of its edge weights[5]. A binary random network was generated by randomly placing *m* edges amongst *n* nodes, with *m* and *n* being the number of edges and nodes in the observed network, respectively[6]. A weighted random network was then generated by randomly allocating edge weights of the observed network on the edges of the binary random network. For each yearly observed network, 5000 random networks were simulated. A p-value was defined as the proportion of random networks in which the average path length (or clustering

coefficient) was equal to or higher than the average path length (or clustering coefficient) of the corresponding observed network.

The following centrality measures were computed using the igraph[2] package: in- and out-degree, in- and out-strength, betweenness and closeness. In- and out-degree were highly correlated with in- and out-strength, which accounted for the number of livestock movements. This study, therefore, presented in- and out-strength only. Closeness and betweenness measured how close a commune was from others and the extent to which a given commune was laid on the shortest path between two other communes, respectively. While edge weight was accounted for both measures, edge direction was accounted only for betweenness.

References

1 R Core Team (2013). R: A language and environment for statistical computing. R Foundation for Statistical Computing, Vienna, Austria. URL www.R-project.org/

2 Csardi, G. & Nepusz, T. The igraph software package for complex network research. *InterJournal* Complex Systems, 1695 (2006).

3 Opsahl, T. Structure and Evolution of Weighted Networks. *University of London (Queen Mary College), London, UK*, 104-122 (2009).

4 Dijkstra, E. W. A note on two problems in connexion with graphs. *Numer. Math.* **1**, 269-271, doi:10.1007/bf01386390 (1959).

5 Opsahl, T. & Panzarasa, P. Clustering in weighted networks. *Social Networks* **31**, 155-163, doi:https://doi.org/10.1016/j.socnet.2009.02.002 (2009).

6 Butts, C. T. sna: Tools for Social Network Analysis (2016).

Supplementary Table S1a. The association between in-strength and livestock or human population size

Yearly network (July – June)	In-strength			
	Livestock population		Human population	
	Coefficient*	P-value†	Coefficient*	P-value†
2007 - 2008	0.25	0.456	-0.01	0.723
2008 - 2009	0.95	0.168	0.03	0.599
2009 - 2010	0.34	0.472	0.00	0.978
2010 - 2011	0.48	0.270	0.00	0.997
2011 - 2012	0.69	0.019	0.02	0.371
2012 - 2013	0.54	0.175	0.00	0.942
2013 - 2014	0.37	0.143	0.00	0.798

* The value of 1 indicates that in-strength increases by 1 as livestock or human population increases by 100.
† Two-tailed p-values from a permutation test

Supplementary Table S1b. The association between out-strength and livestock or human population size

Yearly network (July – June)	Out-strength			
	Livestock population		Human population	
	Coefficient*	P-value†	Coefficient*	P-value†
2007 - 2008	1.40	0.003	0.09	0.050
2008 - 2009	3.32	<0.001	0.22	0.023
2009 - 2010	1.63	0.002	0.07	0.178
2010 - 2011	1.40	0.013	0.05	0.490
2011 - 2012	1.07	0.016	0.04	0.429
2012 - 2013	1.23	0.038	0.04	0.540
2013 - 2014	1.23	0.002	0.07	0.055

* The value of 1 indicates that in-strength increases by 1 as livestock or human population increases by 100.
† Two-tailed p-values from a permutation test

Supplementary Table S2. The association between the number of livestock movements and road distance between communes

Yearly network (July – June)	Road distance	
	Coefficient*	P-value
2007 - 2008	-0.07	<0.001
2008 - 2009	-0.13	<0.001
2009 - 2010	-0.09	<0.001
2010 - 2011	-0.08	<0.001
2011 - 2012	-0.06	<0.001
2012 - 2013	-0.09	<0.001
2013 - 2014	-0.06	<0.001

*The value of 1 indicates that the number of livestock movements increases by 1 as the road distance between communes increases by 1 km.

Supplementary Table S3a. The median and relative time from disease incursion to commune infection, accounting for communes not infected during simulations (p_{inf} =0.1)

Commune	Class	Median time-to-infection*			Relative time-to-infection †		
		Original	Lower	Upper	Original	Lower	Upper
Sada	Central	336	344	346	1.00	1.00	1.00
Tsingoni	Central	341	346	347	1.00	1.01	1.00
Ouangani	Central	358	364	364	1.07	1.06	1.05
Dembeni	Central	369	381	383	1.10	1.11	1.11
Mamoudzou	Central	403	413	416	1.20	1.20	1.20
Chirongui	Outer	412	436	443	1.23	1.27	1.28
Kani-Keli	Outer	466	477	485	1.39	1.39	1.40
Bandraboua	Outer	452	486	501	1.35	1.41	1.45
Pamandzi	Outer	427	481	501	1.27	1.40	1.45
Chiconi	Outer	472	497	511	1.40	1.44	1.48
Boueni	Outer	484	493	499	1.44	1.43	1.44
Bandrele	Outer	444	461	467	1.32	1.34	1.35
Dzaoudzi	Outer	484	542	569	1.44	1.58	1.64
Mtsamboro	Outer	598.5	653	703	1.78	1.90	2.03
M'tsangamouji	Outer	596.5	632.5	666	1.78	1.84	1.92
Acoua	Outer	644	699	759	1.92	2.03	2.19
Koungou	Outer	709	726	773	2.11	2.11	2.23

* in days; † commune of Sada was used as a baseline

Supplementary Table S3b. The median and relative time from disease incursion to commune infection, accounting for communes not infected during simulations (p_{inf} =0.5)

Commune	Class	Median time-to-infection*			Relative time-to-infection†		
		Original	Lower	Upper	Original	Lower	Upper
Sada	Central	106	108	108	1.00	1.00	1.00
Tsingoni	Central	114	114	114	1.08	1.06	1.06
Ouangani	Central	121	121	122	1.14	1.12	1.13
Dembeni	Central	130	131	131	1.23	1.21	1.21
Mamoudzou	Central	129	130	130	1.22	1.20	1.20
Chirongui	Outer	137	140	141	1.29	1.30	1.31
Kani-Keli	Outer	138	139	140	1.30	1.29	1.30
Bandraboua	Outer	148	151	152	1.40	1.40	1.41
Pamandzi	Outer	153	158	160	1.44	1.46	1.48
Chiconi	Outer	152	159	161	1.43	1.47	1.49
Boueni	Outer	154	154	155	1.45	1.43	1.44
Bandrele	Outer	158	159	160	1.49	1.47	1.48
Dzaoudzi	Outer	170	175	178	1.60	1.62	1.65
Mtsamboro	Outer	191	193	197	1.80	1.79	1.82
M'tsangamouji	Outer	196	196	198	1.85	1.81	1.83
Acoua	Outer	205	206	208	1.93	1.91	1.93
Koungou	Outer	253	252	259	2.39	2.33	2.40

* in days; † commune of Sada was used as a baseline

Supplementary Table S3c. The median and relative time from disease incursion to commune infection, accounting for communes not infected during simulations (p_{inf} = 0.9)

Commune	Class	Median time-to-infection*			Relative time-to-infection†		
		Original	Lower	Upper	Original	Lower	Upper
Sada	Central	70	72	72	1.00	1.00	1.00
Tsingoni	Central	78	78	78	1.11	1.08	1.08
Ouangani	Central	81	81	81	1.16	1.13	1.13
Dembeni	Central	88	88	88	1.26	1.22	1.22
Mamoudzou	Central	84	84	84	1.20	1.16	1.17
Chirongui	Outer	92	95	95	1.31	1.32	1.32
Kani-Keli	Outer	92	93	93	1.31	1.29	1.29
Bandraboua	Outer	98	100	101	1.40	1.38	1.40
Pamandzi	Outer	107	111	112	1.53	1.54	1.56
Chiconi	Outer	99	105	106	1.41	1.46	1.47
Boueni	Outer	103	103	103	1.47	1.43	1.43
Bandrele	Outer	108	110	110	1.54	1.53	1.53
Dzaoudzi	Outer	114	116	116	1.63	1.61	1.61
Mtsamboro	Outer	130	132	134	1.86	1.83	1.86
M’tsangamouji	Outer	132	131.5	132	1.89	1.83	1.83
Acoua	Outer	147	149	149	2.10	2.06	2.07
Koungou	Outer	168	169	173	2.40	2.35	2.40

* in days; † commune of Sada was used as a baseline

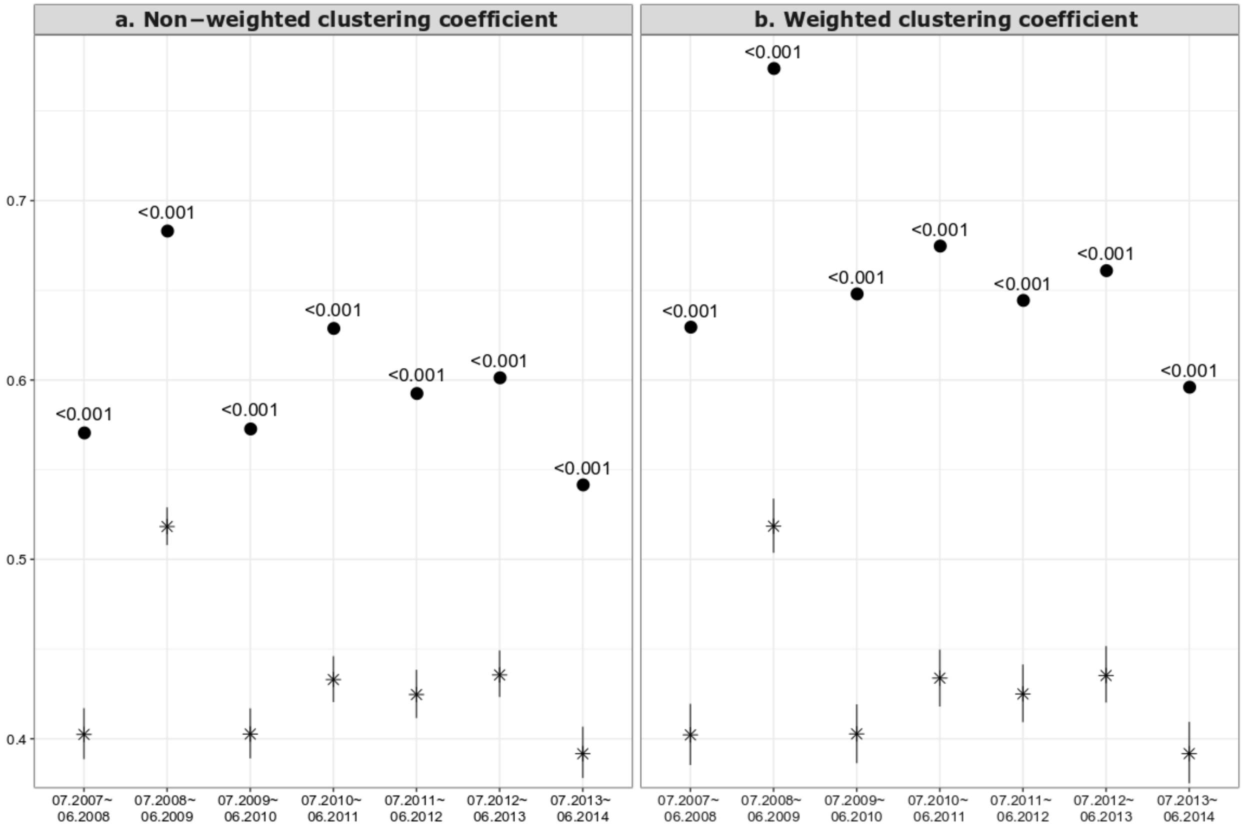

Supplementary Figure S1. Clustering coefficient of the non-weighted (a) and weighted networks (b) for the seven annual networks

The dots represent the clustering coefficient of the observed networks. The crosses and vertical bars, are the median, the first and the third quartiles values obtained from 5,000 random networks. The numbers above the dots indicate the one-sided p-values.

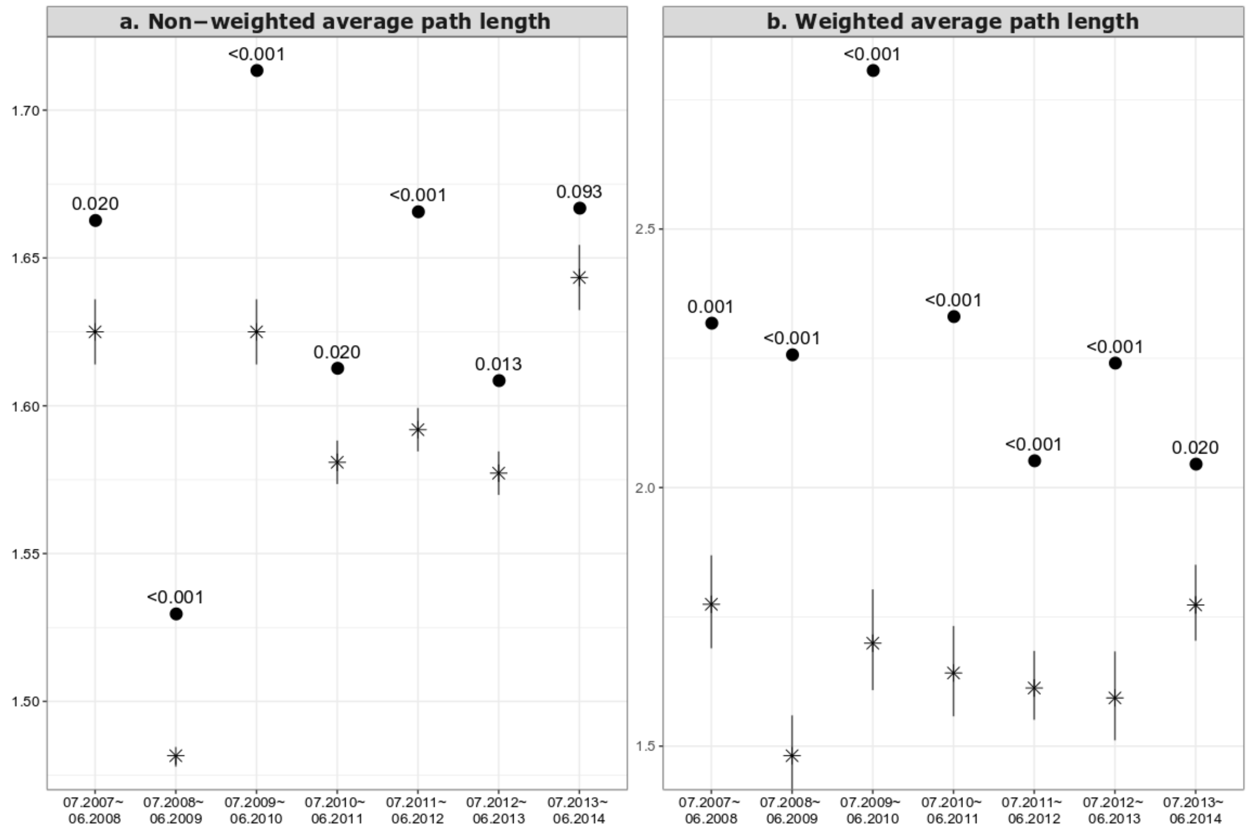

Supplementary Figure S2. Average path length expressed as the mean number of livestock movements between two communes, for the non-weighted (a) and weighted networks (b) for the seven annual networks

The dots represent the average path length of the observed networks. The crosses and vertical bars, are the median, the first and the third quartiles values obtained from 5,000 random networks. The numbers above the dots indicate the one-sided p-values.

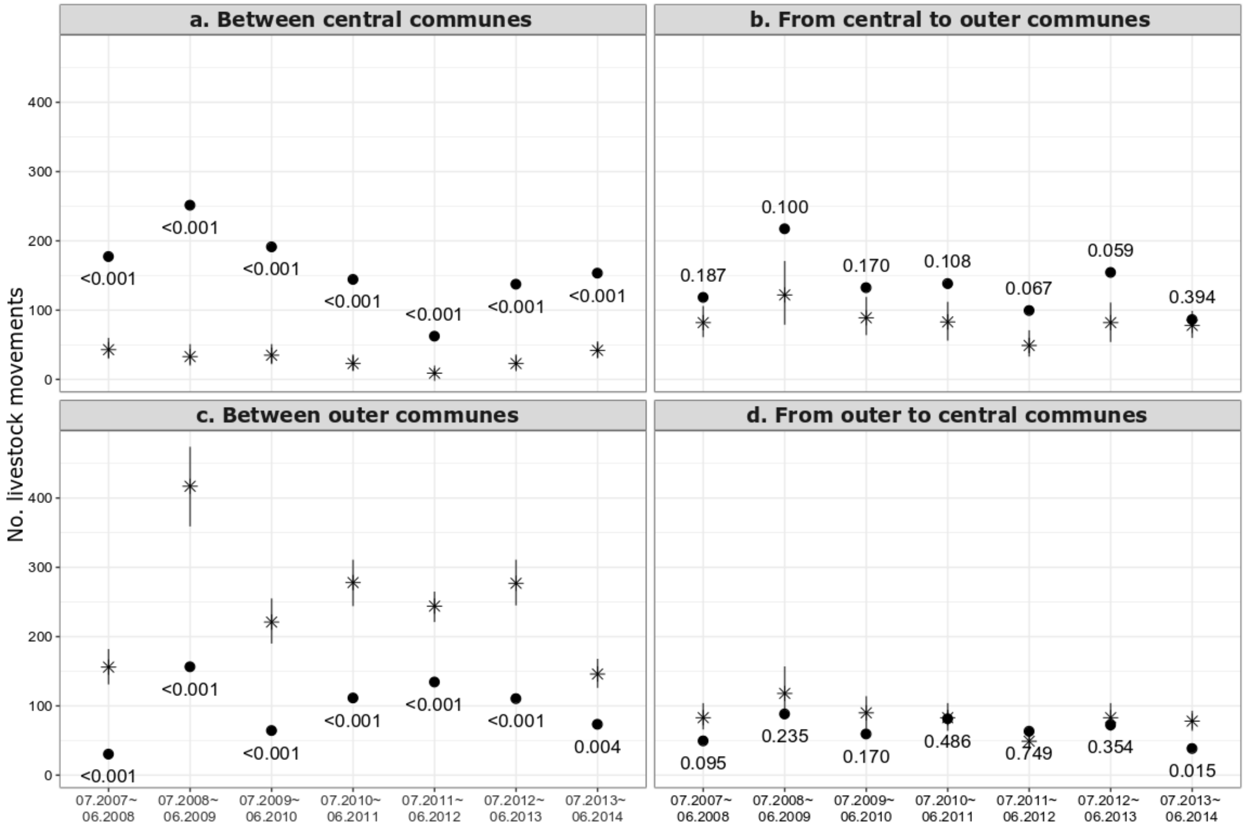

Supplementary Figure S3. Number of livestock movements within and between structurally equivalent classes: (a) between central communes, (b) from central to outer communes, (c) between outer communes and (d) from outer to central communes.

The dots represent the measures of the observed networks, and the vertical lines express those of 5,000 permuted networks; the asterisks corresponded to the median, and the low and high ends corresponded to the first and third quartile. The numbers below or above the dots indicate the one-sided permutation p-values.

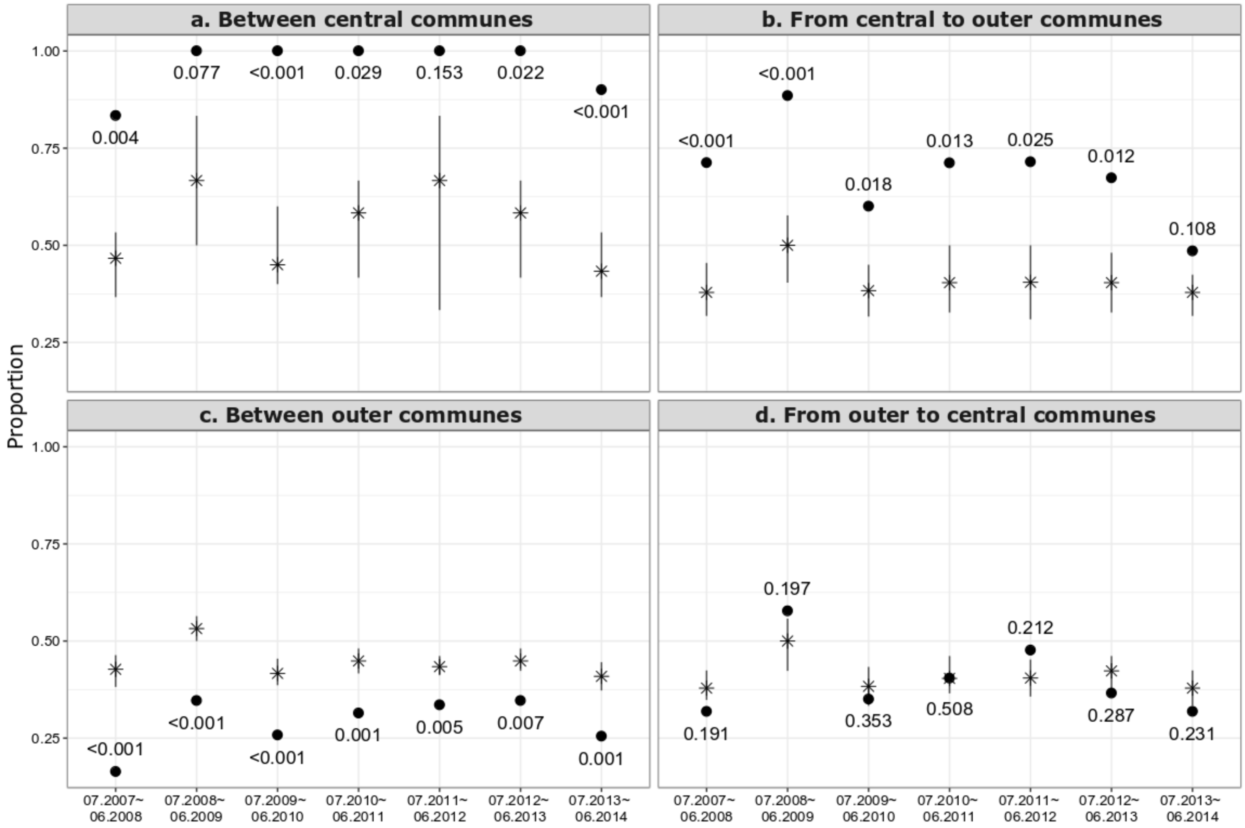

Supplementary Figure S4. Proportion of potential edges that existed within and between structurally equivalent classes: (a) between central communes, (b) from central to outer communes, (c) between outer communes and (d) from outer to central communes

The dots represent the measures of the observed networks, and the vertical lines express those of 5,000 permuted networks; the asterisks corresponded to the median, and the low and high ends corresponded to the first and third quartile. The numbers below or above the dots indicate the one-sided permutation p-values.

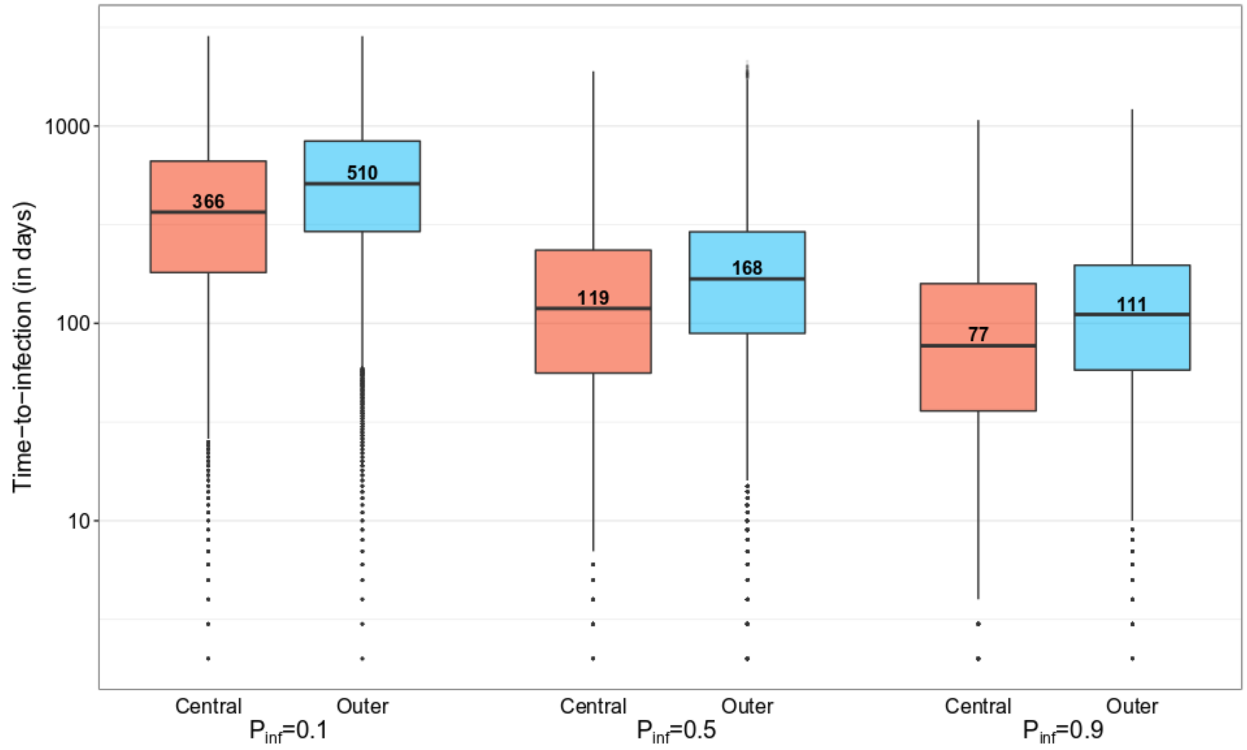

Supplementary Figure S5. Time from disease incursion to commune infection (in days) aggregated by structurally equivalent cluster from the model accounting for potential recall bias in the reported movement dates

For the three values of p_{inf} tested (0.1, 0.5, 0.9), the number of days from disease incursion to commune infection was aggregated by structurally equivalent cluster (central and outer communes) and plotted in the log scale (y-axis). Numbers in boxplots corresponded to the median values.

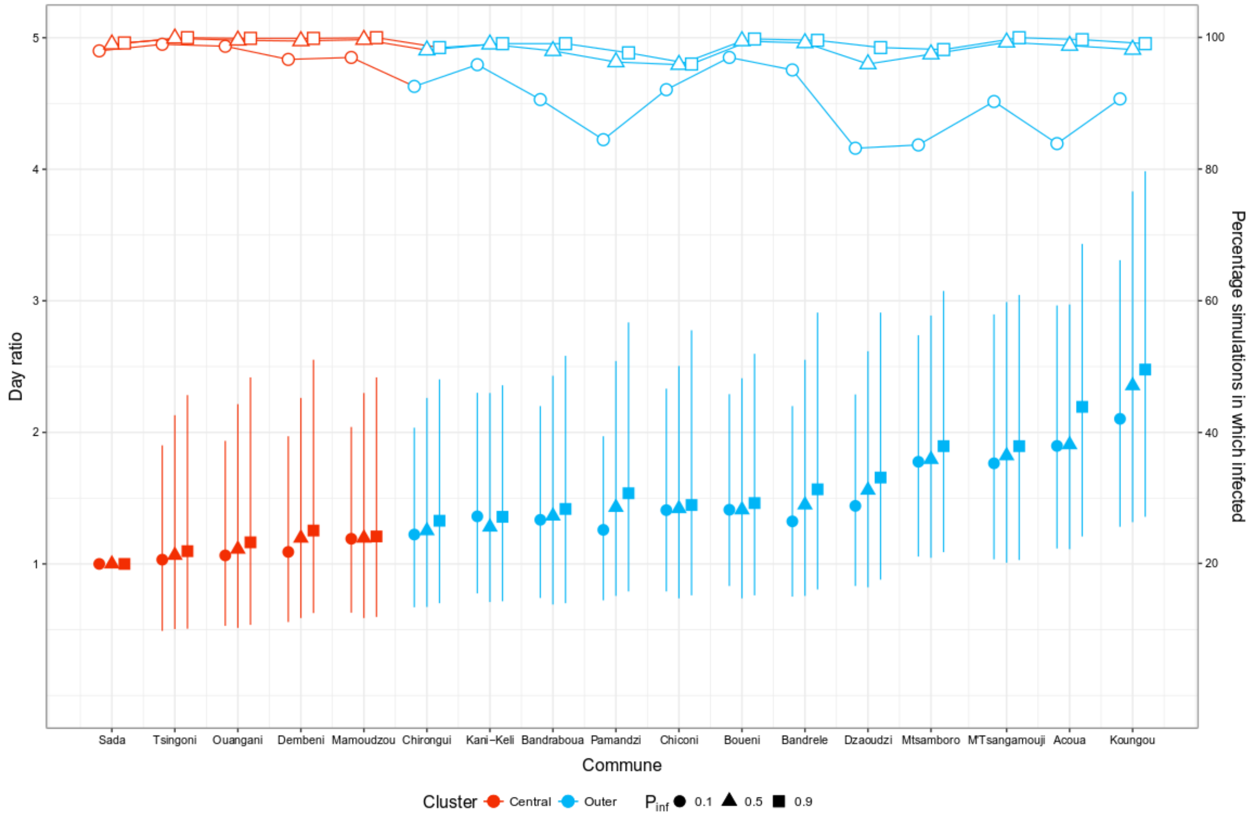

Supplementary Figure S6. Relative time from disease incursion to infection of individual communes from the model accounting for potential recall bias in the reported movement dates.

The time from disease incursion to commune infection was compared for each commune using the commune of Sada as a baseline. The median, and 1st and 3rd quartiles of individual communes were compared with the median of Sada and presented by filled points and vertical lines (right x-axis). For each commune, the percentage of simulations in which the commune was infected was presented by hollow points and horizontal lines (right y-axis). Different point shapes and colours represented different p_{inf} values and structurally equivalent clusters, respectively.

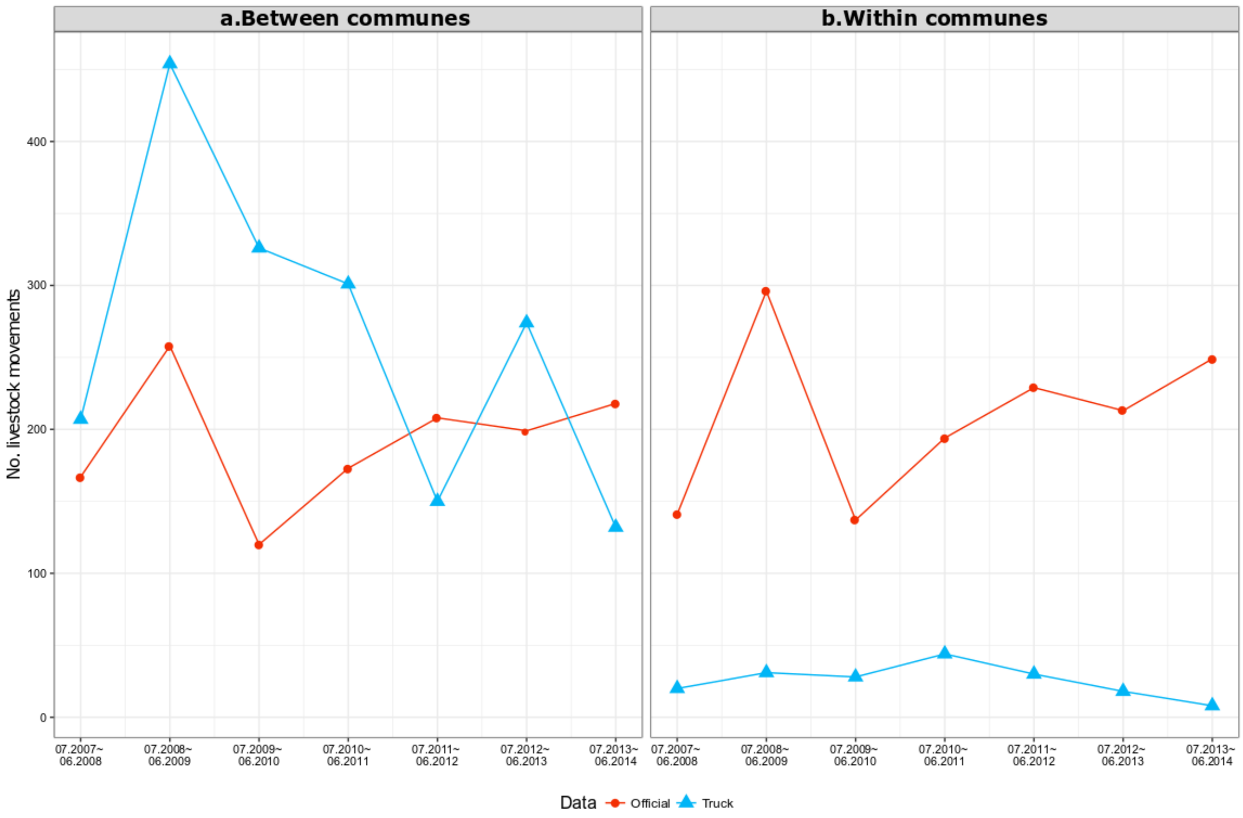

Supplementary Figure S7. Number of annual livestock movements (a) between communes and (b) within communes, recorded in the official and the truck datasets
